# Meta-analysis of Alzheimer’s disease on 9,751 samples from Norway and IGAP study identifies four risk loci

**DOI:** 10.1038/s41598-018-36429-6

**Published:** 2018-12-27

**Authors:** Aree Witoelar, Arvid Rongve, Ina S. Almdahl, Ingun D. Ulstein, Andreas Engvig, Linda R. White, Geir Selbæk, Eystein Stordal, Fred Andersen, Anne Brækhus, Ingvild Saltvedt, Knut Engedal, Timothy Hughes, Sverre Bergh, Geir Bråthen, Nenad Bogdanovic, Francesco Bettella, Yunpeng Wang, Lavinia Athanasiu, Shahram Bahrami, Stephanie Le Hellard, Sudheer Giddaluru, Anders M. Dale, Sigrid B. Sando, Stacy Steinberg, Hreinn Stefansson, Jon Snaedal, Rahul S. Desikan, Kari Stefansson, Dag Aarsland, Srdjan Djurovic, Tormod Fladby, Ole A. Andreassen

**Affiliations:** 1 NORMENT, KG Jebsen Centre for Psychosis Research, Division of Mental Health and Addiction, Oslo University Hospital, Oslo, Norway; 2 Institute of Clinical Medicine, University of Oslo, Oslo, Norway; 3 Department of Molecular Medicine, University of Oslo, Oslo, Norway; 4 Department of Research and Innovation, Helse Fonna, Haugesund, Norway; 5 Department of Clinical Medicine, University of Bergen, Bergen, Norway; 6 Department of Neurology, Akershus University Hospital, Lørenskog, Norway; 7 University of Oslo, AHUS Campus, Oslo, Norway; 8 Department of Psychiatry of Old Age, Oslo University Hospital, Oslo, Norway; 9 Department of Internal Medicine, Oslo University Hospital, Oslo, Norway; 10 Department of Neuromedicine and Movement Science, Norwegian University of Science and Technology, Trondheim, Norway; 11 Department of Neurology, St Olav’s Hospital, Trondheim University Hospital, Trondheim, Norway; 12 Norwegian National Advisory Unit on Ageing and Health, Vestfold Hospital Trust, Tønsberg, Norway; 13 Institute of Health and Society, University of Oslo, Oslo, Norway; 14 Department of Psychiatry, Namsos Hospital, Namsos, Norway; 15 Department of Mental Health, Norwegian University of Science and Technology, Trondheim, Norway; 16 Department of Community Medicine, University of Tromsø, Tromsø, Norway; 17 Geriatric Department, University Hospital Oslo and University of Oslo, Oslo, Norway; 18 Department of Geriatrics, St. Olav’s Hospital, Trondheim University Hospital, Trondheim, Norway; 19 Department of Medical Genetics, Oslo University Hospital, Oslo, Norway; 20 Centre for Old Age Psychiatry Research, Innlandet Hospital Trust, Ottestad, Norway; 21 NORMENT, KG Jebsen Centre for Psychosis Research, Department of Clinical Science, University of Bergen, Bergen, Norway; 22 Dr. Einar Martens Research Group for Biological Psychiatry, Center for Medical Genetics and Molecular Medicine, Haukeland University Hospital, Bergen, Norway; 23 Neuroradiology Section, Department of Radiology and Biomedical Imaging, University of California, San Francisco, San Francisco, CA USA; 24 Departments of Cognitive Sciences, University of California, San Diego, La Jolla, CA USA; 25 Departments of Neurosciences, University of California, San Diego, La Jolla, CA USA; 26 Department of Radiology, University of California, San Diego, La Jolla, CA USA; 27 deCODE Genetics, Reykjavik, Iceland; 28 Landspitali University Hospital, Department of Geriatrics, Reykjavik, Iceland; 29 Institute of Psychiatry, Psychology and Neuroscience, King’s College London, London, UK; 30 Center for Age-Related Diseases, Stavanger University Hospital, Stavanger, Norway

## Abstract

A large fraction of genetic risk factors for Alzheimer’s Disease (AD) is still not identified, limiting the understanding of AD pathology and study of therapeutic targets. We conducted a genome-wide association study (GWAS) of AD cases and controls of European descent from the multi-center DemGene network across Norway and two independent European cohorts. In a two-stage process, we first performed a meta-analysis using GWAS results from 2,893 AD cases and 6,858 cognitively normal controls from Norway and 25,580 cases and 48,466 controls from the International Genomics of Alzheimer’s Project (IGAP), denoted the discovery sample. Second, we selected the top hits (p < 1 × 10^−6^) from the discovery analysis for replication in an Icelandic cohort consisting of 5,341 cases and 110,008 controls. We identified a novel genomic region with genome-wide significant association with AD on chromosome 4 (combined analysis OR = 1.07, p = 2.48 x 10^-8^). This finding implicated *HS3ST1*, a gene expressed throughout the brain particularly in the cerebellar cortex. In addition, we identified *IGHV1-68* in the discovery sample, previously not associated with AD. We also associated *USP6NL/ECHDC3* and *BZRAP1-AS1* to AD, confirming findings from a follow-up transethnic study. These new gene loci provide further evidence for AD as a polygenic disorder, and suggest new mechanistic pathways that warrant further investigation.

## Introduction

Alzheimer’s disease (AD), the most common cause of dementia^1,2^, places a large personal and economic burden on families and society^3,4^. There are nearly 200,000 individuals suffering from AD in the Nordic countries alone (http://www.alzheimer-europe.org). In the absence of disease-modifying therapies, identifying AD prevention strategies is of importance. Converging evidence indicates that AD-associated pathological changes^5^ begin years, if not decades, before the onset of clinical symptoms^6–8^. In order for targeted prevention to be most effective, there is a need to better identify the genetic AD architecture to determine pre-symptomatic disease risk, as well as to provide new insight into disease mechanisms.

Multiple genes in combination with environmental risk factors affect AD neurodegeneration^9,10^. Apart from the major genetic risk factor of the *APOE* gene^11–13^, late onset AD does not exhibit a clear-cut pattern of inheritance and is probably caused by many common variants, each with a small effect^9,14–19^ (‘polygenic’), together with few rare variants with large effects^9,19–22^. In a genome-wide association study (GWAS), SNPs tagging these causal variants may be identified as association loci. In a large, two-stage GWAS meta-analysis^15^, 19 loci were associated with AD in addition to the previously reported APOE locus. Yet these sequence variants account only for a small portion of the disease heritability^23–26^. This “missing heritability” has been attributed to a number of potential causes, such as lack of typing of rare variants^26^, and lack of proper statistical methods for analyzing the polygenic architecture of AD. A concerted effort to increase sample sizes is required to reveal the small-effect genetic risk variants which are common in the population.

We conducted a GWAS of 2,893 AD cases and 6,858 controls from a Norwegian cohort and meta-analyzed this cohort with the publicly available IGAP GWAS data^15^. The most promising association signals were tested in an independent replication sample from Iceland. A novel locus was uncovered on chromosome 4 (rs6448807; hg19 chr4:11676144, OR = 1.07, p = 2.48 × 10^−8^). This novel locus is close to the *HS3ST1* gene, suggesting the involvement of a new biochemical pathway in AD pathology.

## Methods

### Samples

#### Norwegian cohorts

We collected genotype data from the Norwegian DemGene network consisting of 2,893 cases and 1,660 healthy controls. A total of 4,553 Caucasian (1000 Genome population code: CEU) individuals were recruited and successfully genotyped. DemGene is a Norwegian network of clinical sites collecting cases from Memory Clinics based on standardized examination of cognitive, functional and behavioral measures and data on progression of most patients. We diagnosed 2,135 cases of AD dementia as well as 758 cases of Mild Cognitive Impairment (MCI) as a proxy of prodromal AD, from 7 studies (mean age = 73.2 ± 9.9): the Norwegian Register of persons with Cognitive Symptoms (NorCog), the Progression of Alzheimer’s Disease and Resource use (PADR), the Dementia Study of Western Norway (DemVest), the AHUS study, the Dementia Study in Rural Northern Norway (NordNorge), HUNT Dementia Study and the Nursing Home study, the TrønderBrain study, and the Dementia Disease Initiation study (DDI). Cases were diagnosed with dementia due to Alzheimer’s disease according to the recommendations from the National Institute on Aging–Alzheimer’s Association (NIA/AA) (AHUS), the NINCDS-ADRDA criteria (DemVest and TrønderBrain) or the ICD-10 research criteria (NorCog, PADR, NordNorge and HUNT). MCI was diagnosed according to the NIA-AA criteria (AHUS and DDI) or the Winblad criteria (NorCog, HUNT, PADR, DemVest, Trønderbrain). The controls from Norway were obtained through AHUS, NordNorge, HUNT and TrønderBrain studies. Controls were screened with standardized interview and cognitive tests from the population. To increase the statistical power of our association analysis, the controls were combined with additional 4,475 population controls from Norwegian blood donor samples (Oslo University Hospital, Ullevål Hospital, between 18–60 years) and 723 controls from the Thematically Organized Psychosis Research (TOP) Study^27^ (between 25–65 years). Control subjects from the TOP Study were of Caucasian origin without history of moderate/severe head injury, neurological disorder, mental retardation and were excluded if they or any of their close relatives had a lifetime history of a severe psychiatric disorder, a history of medical problems thought to interfere with brain function or significant illicit drug use^27^. The inclusion of population controls were corrected for population stratification^28^. For a complete description of the samples, please refer to Supplementary Text 1 and Supplementary Table 1.

#### Genotyping

The genotypes for the DemGene Study were obtained with Human Omni Express-24 v.1.1 (Illumina Inc., San Diego, CA, USA) at deCODE Genetics (Reykjavik, Iceland).

#### IGAP Study

We obtained summary statistic GWAS data from the IGAP Study, a large two-stage meta-analysis of GWAS in individuals of European ancestry. In stage 1, genotyped data were imputed into 7,055,881 SNPs to perform meta-analysis on 4 previously published GWAS data sets from four consortia: the Alzheimer’s Disease Genetic Consortium (ADGC), the Cohorts for Heart and Aging Research in Genomic Epidemiology (CHARGE) Consortium, the European Alzheimer’s Disease Initiative (EADI) and the Genetic and Environmental Risk in Alzheimer’s Disease (GERAD) Consortium, consisting of 17,008 AD cases and 37,154 controls. In stage 2, 11,632 SNPs were genotyped and tested for association in an independent set of 8,572 AD cases and 11,312 controls.

#### Sample from Iceland

Patients from Iceland were diagnosed with definite, probable or possible AD dementia on the basis of the NINCDS-ADRDA criteria or according to guidelines for ICD-10 F00 and were compared to population controls^17^. Association testing was carried out using information from 5,341 AD patients and 110,008 population controls, adjusting for age, age-squared, sex and county of birth.

The case-control studies are summarized in Table 1.

**Table 1:** Samples.

	Cases (MCI)	Percent Women	Age	Controls
DemGene*	2,893 (758)	60	73.2 ± 9.9	6,858
IGAP				
-Stage 1	17,008	61.3	74.2	37,154
-Stage 2	8,572	64.7	74.4	11,312
Discovery	28,473			55,324
Replication	5,341			110,008

^*^Cases include Alzheimer’s Disease (AD) dementia and Mild Cognitive Impairment (MCI). IGAP and replication cases include AD dementia only.

### Analysis

Before imputation, the genotype data underwent basic quality control including removal of SNPs with minor allele frequency (MAF) lower than 0.01, genotype call rate lower than 0.95, less than one in a million probability of being in Hardy-Weinberg equilibrium (for both cases and controls) and ambiguous strand assignment (A/T, C/G SNPs). The quality-controlled genotypes were used to compute genetic principal components analysis (PCA) with which to adjust for potential population stratification effects. PCA was performed using PLINK 1.9 based on the variance-standardized relationship matrix^29^.

MaCH software^30^ was used to impute the genotypes of all participants onto reference haplotypes derived from samples of European ancestry in the 1000 Genome Project (Build 37, Assembly Hg19)^31^. SNPs with MAF lower than 0.005 or a ratio of observed versus expected variance lower than 0.1 were excluded. The association of LOAD with genotype dosage was analyzed in PLINK 1.9 with a logistic regression model using gender and 20 top principal components as covariates. Sex chromosomes were not included from the analyses^29^. After filtering for SNPs with information value greater than 0.5, we obtained 9,155,276 association p-values. The inflation factor of the DemGene sample was calculated at 1.022 (Supplementary Fig. 1).

We analyzed 6,564,314 SNPs that overlap between DemGene and IGAP stage 1 data and 10,092 SNPs with IGAP stage 2. A meta-analysis with IGAP data was performed using PLINK 1.9 with fixed effects inverse-variance weighted effect sizes^29^.

### Functional Annotation (FUMA)

We utilized FUMA for post-processing of our Stage 1 results^32^. FUMA incorporates 18 biological data repositories such as the Genotype-Tissue Expression (GTEx), Encyclopedia of DNA Elements (ENCODE), Roadmap Epigenomics Project and chromatin interaction information. FUMA requires GWAS summary statistics and outputs multiple tables and figures containing extensive information on, e.g., functionality of SNPs in genomic risk loci, including protein-altering consequences, gene-expression influences, open-chromatin states as well as three-dimensional (3D) chromatin interactions. Functionally annotated SNPs are subsequently mapped to prioritized genes based on (i) physical position mapping on the genome, (ii) expression quantitative trait loci (eQTL) mapping and (iii) 3D chromatin interactions (chromatin interaction mapping)^32^. Biological information for each prioritized gene is provided to gain insight into previously associated diseases. Beside the single gene level analyses, FUMA uses GTEx to identify 53 tissue specific expression levels of prioritized genes. We refer to the original publication for details on the methods and repositories of FUMA^32^.

## Results

**Figure 1:**
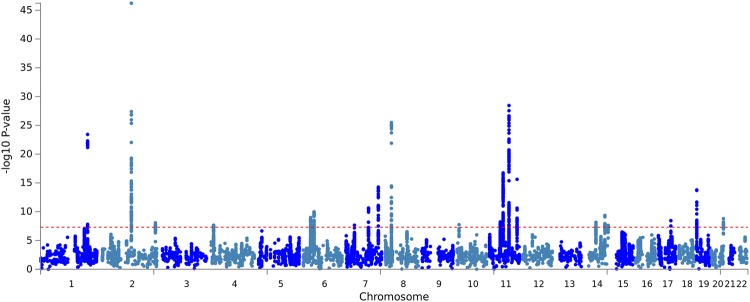
Manhattan plots of discovery sample from meta-analysis of DemGene data with IGAP 1 + 2. Note that color alternate between chromosomes for visualization only. Out of the loci passing genome-wide threshold p < 5 × 10^−8^, we found novel hits on Chromosomes 4, 10, 14 and 17.

We performed GWAS in two stages^33,34^. First, we meta-analyzed the discovery samples using 10,092 SNPs imputed for both the DemGene and IGAP stage 1 + 2 samples (Manhattan plots in Fig. 1). From this discovery sample result, we narrowed our analysis to 1,035 SNPs with genome-wide or suggestive association (meta p < 1 × 10^−6^) and tested these SNPs in an Icelandic replication sample. Finally, we meta-analysed the discovery and replication samples into a combined analysis.

We identified 20 genomic risk loci from the discovery sample using FUMA (r^2^ > 0.1 using Linkage Disequilibrium from 1000 Genomes hg19^31^), listed in Supplementary Table 2. Four out of the 20 loci had not reached genome-wide significance in IGAP at p < 5 × 10^−8^. We list the lead SNPs of the discovery sample in Table 2 and the corresponding lead SNPs of the combined analysis in Supplementary Table 3.

**Figure 2:**
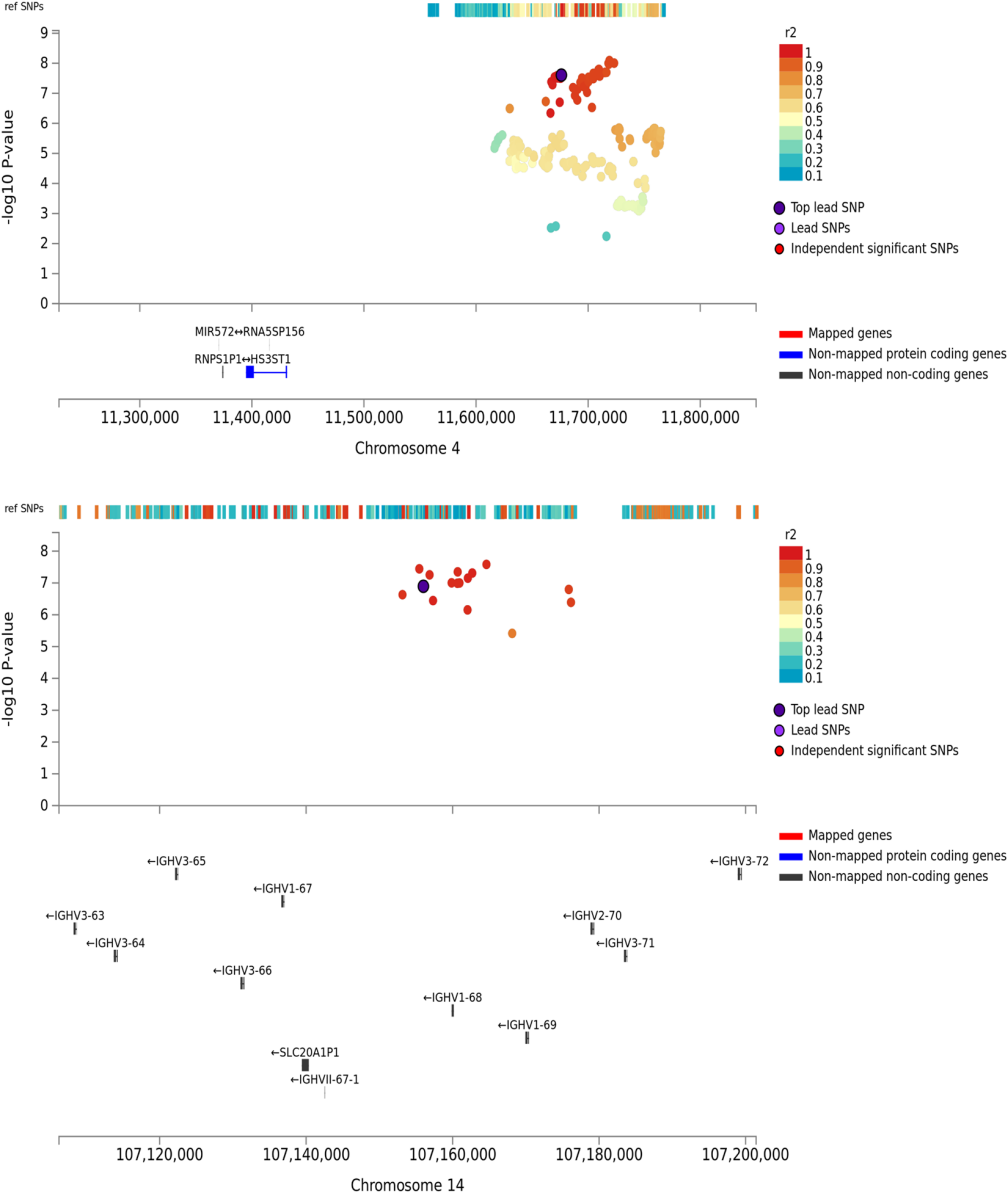
Regional association plots of rs6448807 (chr 4) and rs79452530 (chr 14) in the discovery sample. Mapping of the genes is strictly based on position of the genomic risk loci.

We highlight a genomic region on chromosome 4 spanning 11.55 Mb–11.76 Mb (best SNP: rs6448807, discovery OR = 1.08, p = 2.23 × 10^−8^) in Fig. 2. The lead SNP is located at position chr4:11676144 (minor/major allele: C/T), an intron of gene *RP11-281P23*.*2*, a non-coding RNA (Ensembl: *ENSG00000249631)*. Its nearest protein-coding gene is *HS3ST1* located 240 kb away. We found the same direction of effects within the replication sample (replication OR = 1.03, p = 0.17), and in the combined analysis the SNP is associated with AD at p = 2.48 × 10^−8^ (Table 2). Although this lead SNP of the discovery sample was not replicated at p < 0.05 in the replication sample, we found rs13133131 within the same genomic locus as a proxy SNP (r^2^ = 0.94) with better replication (replication p = 0.00319) and a stronger association in the combined analysis (combined OR = 1.07, p = 8.16 × 10^−9^), see Supplementary Table 3 for genomic risk loci based on the combined analysis.

**Table 2:** Genomic risk loci from the discovery sample which are not genome-wide significant in IGAP.

CHR	BP	SNP	A1/A2	MAF	Gene	Discovery		Replication		Combined		Dir.
						OR	p-value	OR	p-value	OR	p-value	
4	11676144	rs6448807	T/C	0.30	*RP11-281P23*.*2* *(HS3ST1)*	1.08	2.23E-08	1.03	1.72E-01	1.07	2.48E-08	+++
10	11720308	rs7920721	G/A	0.27	*RP11-138I18*.*2* *(USP6NL*/*ECHDC3)*	1.07	1.82E-08	1.06	7.23E-03	1.07	4.84E-10	+++
14	107156009	rs79452530	T/C	0.16	*IGHV1-68* *(IGHV1-68)*	0.89	2.36E-08	0.97	3.08E-01	0.91	1.27E-07	—
17	56404349	rs2526378	G/A	0.47	*BZRAP1-AS1* *(BZRAP1)*	0.93	3.64E-09	0.99	6.69E-01	0.94	5.30E-08	—

CHR: chromosome; BP: base pair location; A1/A2: major/minor alleles; MAF: minor allele frequency in 1000 G. Gene: nearest gene and nearest protein-coding gene (in parentheses). OR: odds ratio; Dir.: direction of effects in the two discovery samples (Demgene, IGAP) and the replication sample (Iceland).

Additionally we found a genomic region on chromosome 14 spanning 106.47 Mb–107.26 Mb with a novel risk associated locus at rs79452530 (chr14:107156009, OR = 0.89, p = 2.36 × 10^−8^), see Fig. 2. This is an intergenic SNP 3,860 kb away from the nearest gene *IGHV1-68* (Ensembl: ENSG0000253703). The locus has not previously been associated with AD, but was mentioned in a study in rheumatic heart disease^35^. This genomic region was genome-wide significant in the discovery sample, but its effect was not observed in the replication sample (Table 2) and none of its proxies passed the significance threshold in the combined analysis (best SNP: rs78631692 at p = 6.57 × 10^−8^, Supplementary Table 3). The directions of effects, however, were consistent in all samples (Table 2).

We also found association regions at genome-wide significance at two additional loci on Chromosome 10 and 17, both of which had not passed significance threshold in the IGAP study. The first locus, rs7920721 (chr10:11720308, OR = 1.07, p = 1.82 × 10^−8^), is an intergenic SNP with nearest gene *RP11-138I18*.*2* (Ensembl: *ENSG00000271046*) at 1366 kb away and in between protein-coding genes *USP6NL/ECHDC3* (see Supplementary Fig. 2). This SNP is replicated at p = 0.0072 and has a strong association signal in the combined analysis (OR = 1.07, p = 4.84 × 10^−10^). The second locus, SNP rs2526378 (chr17:56404349, OR = 0.93, p = 3.64 × 10^−9^), is an intron of non-coding RNA *BZRAP1-AS1* (Ensembl: *ENSG00000265148*) (Supplementary Fig. 2). A proxy of this SNP (r^2^ = 0.96) is significant in the combined analysis at p = 3.58 × 10^−8^, although it is mainly driven by the discovery sample (Supplementary Table 3). For both rs7920721 and rs2526378, we found agreement of its significance in a following transethnic AD study^36^, confirming our results.

**Figure 3:**
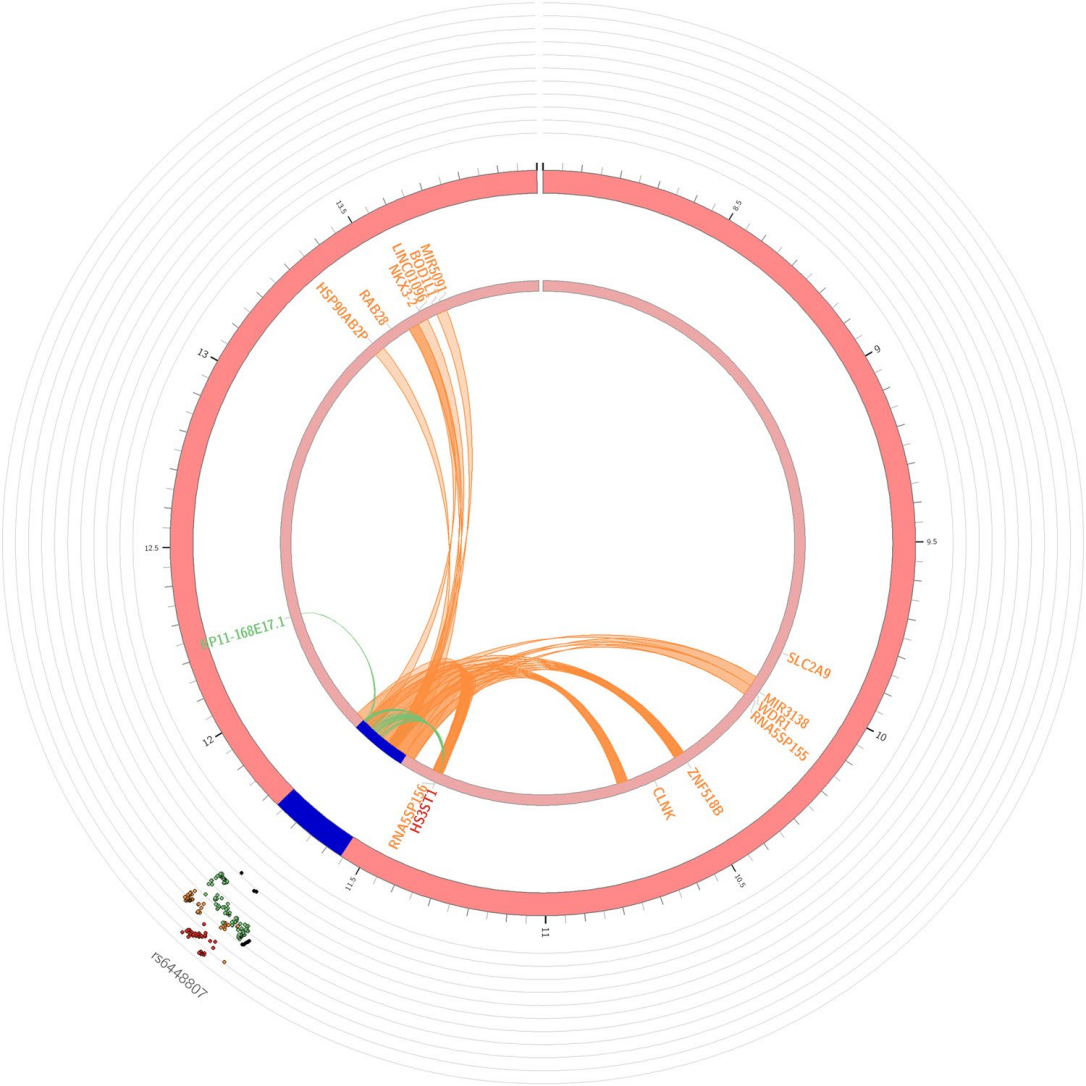
Circos plots of mapped gene on chromosome 4 locus. Genomic risk loci are highlighted in blue. Genes are mapped by 3-D chromatin interaction (orange) or eQTLs (green), or both (red) notably gene *HS3ST1*.

We focused our FUMA analyses on the two novel loci in our study, the two genomic regions on chromosomes 4 and 14, represented by lead SNPs rs6448807 and rs79452530. Out of the functional consequences of 1,128 SNPs represented by the two loci on genes, we found the most SNPs being intergenic (n = 511 SNPs) and non-coding RNA-intronic (n = 340), see Supplementary Fig. 3. For the chromosome 4 locus, we mapped the associated variants through eQTL or 3-D chromatin interaction to 15 genes, eight of which are protein-coding: *BOD1L1*, *CLNK*, *HS3ST1*, *NKX3-2*, *RAB28*, *SLC2A9*, *WDR1* and *ZNF518B* (Fig. 3). Notably, *HS3ST1* was mapping using both eQTL and chromatin interaction. For the chromosome 14 locus, we mapped the associated variants to 117 genes (Supplementary Figs 3 and 4). Despite the large number of genes located in the 780 kb-span of the genomic risk region, none of them was identified as a protein-coding gene by FUMA.

Furthermore, we looked at the expression and enrichment tests of the protein-coding genes prioritized by FUMA. We found *WDR1* to be highly expressed in comparison to other genes in all tissues except brain tissue, where it is only moderately highly expressed (Supplementary Fig. 5). We also found higher expression in *RAB28* and *BOD1L1* in all tissues. Although *HS3ST1* is not highly expressed compared to other genes, its expression is specifically higher in the cerebellar hemisphere and the cerebellum. Lastly, we found *IGHV*-genes to have higher expression in the spleen, stomach, intestine and lung tissues, and lower expression specific to brain tissues.

## Discussion

Our study presents the largest GWAS of AD cases and controls from multi-center network across Norway to date and its meta-analysis with two independent European cohorts. Our main finding in the present study is a novel locus associated with AD on chromosome 4, represented by rs6448807. Its closest protein-coding gene, *HS3ST1*, has been previously suggested as an AD susceptibility locus using conditional False Discovery Rate methods associating AD with dyslipidaemia^37^, and here we discover the same locus using standard GWAS analysis. According to brain expression data from the BrainSpan Atlas of the Developing Human Brain^38,39^, *HS3ST1* (Heparan sulfate D-glucosaminyl 3-O-sulfotransferase 1) is expressed in the brain, with strong expressions in the cerebellar cortex, followed by the primary visual cortex. Similar heparan sulphate enzymes (HST3ST1, HS3ST2, HST3ST3A, HS3ST3B, HS3ST4 and HS3ST5) are expressed in human elderly hippocampus^40^, with HS3ST2 already proven to be overexpressed in the hippocampus of patients with AD^41^. Similarly, heparan sulfates have been shown in mice to modulate brain amyloid-β clearance and aggregation in AD^42^. This study pointed to the implication of heparan sulphates to AD.

In a previous large AD study by IGAP^15^, a proxy to this SNP (r^2^ = 0.79) had been listed as a suggestive association locus (rs6448799; hg19 chr4:11630049). The gene *HS3ST1* is located 197 kb from rs6448799 and 245 kb from rs6448807. This finding adds another risk variant with a low odds ratio to the polygenic risk factors of AD. rs6448807 and rs6448799^15^ are located in the first intron of non-coding RNA *RP11-281P23*. The Ensembl gene model for *RP11-281P23* is supported by expressed sequence tag evidence from testes and kidney, and RNA-seq data provide evidence of expression in the heart, testes^43^ and brain (dorsolateral prefrontal cortex)^44^ although expression levels are low in all these tissues. Based on the available data, it is not possible to conclude on which molecule is driving the association. The genetic evidence would tend to suggest that it is the non-coding RNA that underlies the association given that 1) both the genome-wide significant rs6448807 and the suggestive association locus rs6448799 are located near the transcription start site of the long non-coding RNA, 2) *HS3ST1* is 245 kb away from these loci, and 3) there is little linkage disequilibrium between the *HS3ST1* and the *RP11-281P23* locus. On the other hand, FUMA analyses point to the priority of *HS3ST1* through not only eQTLs but also chromatin interactions. *HS3ST1* has low but consistent expression in the cerebellar and visual cortex^38,39^ as well as the differential expression in brain between AD cases and controls^37^ provide supportive evidence for *HS3ST1* playing an important role. Further experimental research will be required to elucidate the biological basis of the association of this locus with AD.

Furthermore, we have boosted three genomic loci from the previous IGAP study into genome-wide significance. Two of the three loci are confirmed in a following study in transethnic meta-analysis study and here we observed them in a CEU population. The two loci are an intron on a non-coding RNA *BZRAP1-AS1* and an intergenic SNPs with closest gene *RP11-138I18*.*2*.

From the discovery sample, we associated *IGHV1-68* to AD, previously not associated with the disease. *IGHV1-68* is a pseudogene within the immunoglobulin heavy chain (IGH) locus^45^. Although the exact role of *IGHV1-68* is unknown, IGH is essential for biosynthesis of antibodies, a key component of the adaptive immune response^46^. Interestingly, a recent GWAS-study associated *IGHV1-68* with the aberrant immune response associated with rheumatic heart disease^35^. However, the association with AD is novel, and warrants further studies into immunological pathways in AD^47^. A potential weakness of the present study entails the inclusion of younger population controls and the inclusion of MCI cases diagnosed with the Winblad criteria which lack AD biomarker profiling. While the population controls boosted the statistical power in our study, it may include controls that may later develop AD at disease prevalence rate. Thus, this study might not have reached its potential full statistical power. On the inclusion of MCI cases, although not all subjects diagnosed with MCI will ultimately develop AD, a large proportion of those with amnestic MCI and mixed type of MCI are expected to develop AD^48^. The potential inclusion of non-AD MCI cases may have reduced the strength of our findings. However, this only reduces the chance of finding GWAS loci, i.e. likely induce type-II errors but does not affect the rate of type-I errors.

To conclude, our findings further demonstrate the polygenic architecture of AD, and unveil potential mechanisms including the immune system involved in AD pathobiology that warrant further investigation.

## Electronic supplementary material

Supplementary Material

## Data Availability

Genotype datasets from the Norwegian DemGene network generated and analysed during the current study are not publicly available due to compliance to privacy. Summary statistics are available from the corresponding author on reasonable request. The summary statistics from IGAP are publicly available at http://web.pasteur-lille.fr/en/recherche/u744/igap/igap_download.php. Data from Icelandic study are available from deCODE Genetics on reasonable request.
