# Supplementary material for: Meta-analysis of Alzheimer’s disease on 9,751 samples from Norway and IGAP study identifies four risk loci
Source: Sci Rep. 2018 Dec 27;8:18088. doi: 10.1038/s41598-018-36429-6 (PMC6308232; doi:10.1038/s41598-018-36429-6)
Supplement: Supplementary file 1 — Supplementary Material [file 41598_2018_36429_MOESM1_ESM.docx]

**M****eta-analysis of Alzheimer’s disease on 9,751 samples from Norway and IGAP study identifies four risk loci**

Aree Witoelar^1,2,3^, Arvid Rongve^4,5^, Ina S. Almdahl^6,7,8^ , Ingun D. Ulstein^2,8^, Andreas Engvig^1,9^, Linda R. White^10,11^, Geir Selbæk^12,13^, Eystein Stordal^14,15^, Fred Andersen^16^, Anne Brækhus^12,17^, Ingv­ild Saltvedt^10,18^, Knut Engedal^12,17^, Timothy Hughes^1,19^, Sverre Bergh^12,20^, Geir Bråthen^10,11^, Nenad Bogdanovic^17^, Francesco Bettella^1,2^, Yunpeng Wang^1,2^, Lavinia Athanasiu^1,2^, Shahram Bahrami^1,2^, Stephanie Le Hellard^21,22^, Sudheer Giddaluru^21,22^, Anders M. Dale^24,25,26^, Sigrid B. Sando^10,11^, Stacy Steinberg^27^, Hreinn Stefansson^27^, Jon Snaedal^28^, Rahul S. Desikan^23^, Kari Stefansson^27^, Dag Aarsland^7,29,30^ , Srdjan Djurovic^19,21^, Tormod Fladby^6,7^, Ole A. Andreassen*^1,2^

1. NORMENT, KG Jebsen Centre for Psychosis Research, Division of Mental Health and Addiction, Oslo University Hospital, Oslo, Norway
2. Institute of Clinical Medicine, University of Oslo, Oslo, Norway
3. Department of Molecular Medicine, University of Oslo, Oslo, Norway
4. Department of Research and Innovation, Helse Fonna, Haugesund, Norway
5. Department of Clinical Medicine, University of Bergen, Bergen, Norway
6. Department of Neurology, Akershus University Hospital, Lørenskog, Norway
7. University of Oslo, AHUS Campus, Oslo, Norway
8. Department of Psychiatry of Old Age, Oslo University Hospital, Oslo, Norway
9. Department of Internal Medicine, Oslo University Hospital, Oslo, Norway
10. Department of Neuromedicine and Movement Science, Norwegian University of Science and Technology, Trondheim, Norway
11. Department of Neurology, St Olav's Hospital, Trondheim University Hospital, Norway
12. Norwegian National Advisory Unit on Ageing and Health, Vestfold Hospital Trust, Tønsberg, Norway
13. Institute of Health and Society, University of Oslo, Oslo, Norway
14. Department of Psychiatry, Namsos Hospital, Namsos, Norway
15. Department of Mental Health, Norwegian University of Science and Technology, Trondheim, Norway
16. Department of Community Medicine, University of Tromsø, Tromsø, Norway
17. Geriatric Department, University Hospital Oslo and University of Oslo, Oslo, Norway
18. Department of Geriatrics, St. Olav’s Hospital, Trondheim University Hospital, Norway
19. Department of Medical Genetics, Oslo University Hospital, Oslo, Norway
20. Centre for Old Age Psychiatry Research, Innlandet Hospital Trust, Ottestad, Norway
21. NORMENT, KG Jebsen Centre for Psychosis Research, Department of Clinical Science, University of Bergen, Bergen, Norway
22. Dr. Einar Martens Research Group for Biological Psychiatry, Center for Medical Genetics and Molecular Medicine, Haukeland University Hospital, Bergen, Norway
23. Neuroradiology Section, Department of Radiology and Biomedical Imaging, University of California, San Francisco, San Francisco, CA USA
24. Departments of Cognitive Sciences, University of California, San Diego, La Jolla, CA, USA
25. Departments of Neurosciences, University of California, San Diego, La Jolla, CA, USA
26. Department of Radiology, University of California, San Diego, La Jolla, CA, USA
27. deCODE Genetics, Reykjavik, Iceland
28. Landspitali University Hospital, Department of Geriatrics, Reykjavik, Iceland
29. Institute of Psychiatry, Psychology and Neuroscience, King’s College London, UK
30. Center for Age-Related Diseases, Stavanger University Hospital, Stavanger, Norway

Supplementary Text 1. Full Description of Datasets 2

Supplementary Table 1. Sample origins of DemGene 8

Supplementary Table 2. Lead SNPs in the discovery sample 9

Supplementary Table 3. Lead SNPs based on the combined sample 10

Supplementary Figure 1. Quantile-quantile plot of GWAS of Norwegian cohort and meta-analysis with IGAP. 12

Supplementary Figure 2. Regional association plots on chromosome 10 and chromosome 17 13

Supplementary Figure 3. FUMA summary of results 14

Supplementary Figure 4. Chromatin interaction circos plot of chromosome 14 locus 15

Supplementary Figure 5. Gene expression heatmap 16

## Supplementary Text 1. Full Description of Datasets

**Norwegian Study Groups**

All studies were approved by the regional ethics committee and the Norwegian authorities for collection of medical data, and written informed consent was obtained. Although the MCI group included all subtypes and had no biomarkers or cognitive profiles at this stage, few cases with non-amnestic subtype of MCI were recruited into these studies. However, some cases of non-AD pre dementia may still have been included, thereby weakening our findings.

The **AHUS cohort.** Case samples (*N* = 439) were taken from the biobank at Akershus University Hospital in July 2013 and include all samples of EDTA blood contributed to the biobank since 2010 by patients with cognitive complaints referred to lumbar puncture as part of their clinical work-up at the memory clinic. In addition, subjects participating in research projects on mild cognitive impairment and on Parkinson’s disease were included. Control subjects (*N* = 93) without cognitive complaints or known cerebral pathology were included, comprising samples from orthopaedic patients (n=79) and spouses (n=14) of patients with cognitive impairment. *Dementia diagnosis.* The medical records of all participating patients were examined retrospectively and based on all available information patients were diagnosed according to established diagnostic criteria. Biomarkers were used to diagnose mild cognitive impairment due to Alzheimer’s disease according to the “high likelihood” criteria in the NIA/AA-recommendations. MCI cases where no etiological diagnosis could be made with certainty were classified as mild cognitive impairment (MCI), and patients with normal cognitive testing as subjective cognitive impairment (SCI) as previously described^1,2^.

The **COGNORM** study consists of patients scheduled for elective gynaecological (genital prolapse), urological (benign prostate hyperplasia, prostate cancer or bladder tumour/cancer) or orthopaedic (knee or hip replacement) surgery in spinal anaesthesia turning 65 years or older the year of inclusion. They were recruited from Oslo University Hospital and Diakonhjemmet Hospital in Oslo from February 2012 to June 2013. 172 subjects underwent a multi-domain cognitive assessment before the surgery, comprising the Mini Mental Status Examination (MMSE), Clock Drawing Test, Word List Memory Task, Trail Making Test A and B, Kendrick Object Learning Test, and verbal fluency (FAS test and Animal Naming). Informant Questionnaire on Cognitive Decline in the Elderly was filled out by a relative or friend. Blood and CSF were collected in conjunction with anaesthesia, and brain MRI was taken during the months after surgery. Blood samples, CSF samples and brain MRIs were obtained from 163, 155 and 128 subjects, respectively. The subjects were followed with annual cognitive testing since baseline, and at two-year follow up a new brain MRI was obtained from 111 subjects. At the ongoing four-year follow-up a third MRI, an fMRI, and a second lumbar punction for CSF sampling is performed. Annual follow up for five years is planned, making it possible to exclude subjects that develop chronic cognitive impairment.

The **Dementia Disease Initiation** (DDI) Study is a co-operation between all Norwegian health regions and university hospitals, and subjects with self-reported cognitive reduction and healthy controls were recruited from January 2013 till January 2017 and examined following a standard protocol. Anonymized data were collected in a customized database. Recruitment was based on two main sources: 1) Cases were self-referred following advertisements in media, newspapers or news bulletins, or 2) recruited among referrals to local memory clinics. In addition, cognitively healthy controls were also included from spouses of patients with dementia/cognitive disorder, and from patients who completed lumbar puncture for orthopedic surgery. Participants were staged as controls, SCD or MCI applying the NIA-AA criteria were used for cases with lower performance than expected in one or more cognitive domains, but yet preserved independence in functional ability and not fulfilling the criteria of dementia. The controls were further classified as having normal or abnormal cognitive screening and with or without first-degree relative with dementia. Criteria for inclusion were age between 40 and 80 and a native language of Norwegian, Swedish or Danish. Exclusion criteria were brain trauma or disorder, including clinical stroke, dementia, severe psychiatric disease, severe somatic disease that might influence the cognitive functions, or intellectual disability or other developmental disorders. A case report form was developed which included medical history from subject and informant, and physical and neurological examinations including the 15-item Geriatric Depression Score (GDS). The cognitive examination included the Mini Mental State Examination (MMSE-NR), non-verbal cognitive screening (The clock drawing test), verbal memory (CERAD word list), visuoperceptual ability (VOSP silhouettes), psychomotor speed and divided attention (Trail making A and B and word fluency (COWAT). Cognitive functioning was also assessed by the Clinical Dementia Rating scale (CDR). Cases with dementia were excluded if CDR was > 0.5. Lumbar puncture was performed before noon and APOE genotyping was performed. All subjects were referred to an MRI scan.

In the **Dementia Study of Western Norway** (DemVest), all referrals to memory clinics in the Hordaland and Rogaland counties were screened for a first time diagnosis of mild dementia. *Inclusion criteria.* Patients diagnosed for the first time with a dementia diagnosis. To select patients with mild dementia only, all subjects were screened with the Mini-Mental State Examination (MMSE) and a minimal score of 20 and CDR = 1 was required for inclusion. After the initial inclusion period (2005-2007) only DLB or PDD patients were included. *Exclusion criteria.* Patients without dementia or with acute delirium or confusion, terminal illness, recently diagnosed with a major somatic illness, previous bipolar disorder or psychotic disorder were excluded. *Dementia diagnosis*. A research clinician performed a structured clinical interview of demographics, previous diseases, and drug history of patients and caregivers. The assessment procedure included a detailed history using a semi structured interview, clinical examination including physical, neurological, psychiatric, and a detailed neuropsychological test battery, routine blood tests CSF (subgroup), and MRI. Dopamine transporter SPECT was available for most patients with suspect DLB (see ^3^ for details). Patients were followed annually, and diagnoses were re-evaluated with regular intervals by a consensus panel based on all available data. Pathology diagnosis is available for more than 20 cases.

**The HUNT Nord-Trøndelag Dementia Cohort^4^** consists of two populations, a hospital population and a nursing home population. The hospital population recruited cases from the Nord-Trøndelag county hospital dementia register by a two-step procedure. First, a computerized search of the administrative patient database of the two hospitals in Nord-Trøndelag (Namsos Hospital and Levanger Hospital) identified patients with any suspected type of dementia in the period between 1995 and 2010. Second, the hospital records of all such patients were reviewed by a diagnostic team of researches and clinical experts in dementia, consisting of an internist (geriatrician) and two psychogeriatrists. All included cases were diagnosed in two steps, applying first the clinical ICD-10 criteria for dementia, then the research ICD-10 criteria for identifying the AD-cases and vascular dementia (VaD). Dementia with Lewy bodies was diagnosed in cases with two of the following three symptoms: fluctuating cognition, visual hallucinations and motor features of Parkinson’s disease. Cases of frontotemporal dementia had documented changes in behaviour and personality. Cases of other types of dementia met the general criteria for dementia but not the specific criteria for AD, VaD, dementia with Lewy bodies or frontotemporal dementia. The nursing home population recruited cases form a study of all patients in all nursing homes in Nord-Trøndelag County between June 2010 and March 2011. Dementia was assessed by interview with a professional caregiver using a questionnaire for identifying dementia symptoms developed for this study, as well as the Clinical Dementia Rating Scale (CDR). Cognitive function was assessed by two tests: the Mini-Mental-State Examination (MMSE) and the Severe Impairment Battery-8 (SIB-8), and by interview with the relative(s) using the IQ-CODE. In addition, two physicians with wide clinical and research experience independently diagnosed MCI, the dementia syndrome and dementia subtypes, using all available information. More information about the cohort and the method used are described in details in ^4^.

The **Norwegian Register of Cognitive Symptoms** (NorCog) includes patients diagnosed with mild dementia or Mild Cognitive Impairment referred to Memory Clinics. Recruitment began in the South-Eastern part of Norway and is now including most Memory Clinics in Norway. However, only patients from the Memory Clinics at Oslo University Hospital and Innlandet Hospital and Department of geriatrics, St Olav’s hospital are included in the current study. Dementia and cognitive impairment including Alzheimer’s disease are diagnosed according to the ICD-10 research criteria^5^. Patients are home-dwelling outpatients referred to memory clinics, and had standardized comprehensive assessment, including a medical history from the patient as well as a close family member, comprehensive neuropsychological testing, a physical and psychiatric examination with the use of standardized assessment scales, blood sample analyses, CSF when appropriate and structural brain imaging (MRI in most cases, and additionally amyloid-PET in selected cases.

The **Dementia Study in Rural Northern Norway^6^** (NordNorge) recruited patients with recently diagnosed AD in primary health care in nine rural municipalities in Northern Norway from January 2006 to December 2007. AD patients were recruited from general practice (*N* = 87) and through population based screening (*N* = 100). Both groups underwent similar cognitive, physical and laboratory examinations. *Inclusion criteria* were individuals aged ≥ 65 years with a MMSE sum score ≥ 10 points. *Exclusion criteria* were delirium and behavioral disturbances interfering with cognitive and clinical testing, reluctance to participate, and inability to understand the purpose of the study, or relatives/caregivers disapproving participation. Participating clinicians were trained to identify and diagnose AD based on the Norwegian guidelines. In addition to a semi-structured interview of the participants focusing on impairment of cognition and activities of daily living, the Informant Questionnaire on Cognitive Decline in the Elderly (IQ-CODE) was completed, and cognition was tested using The Mini Mental Status Examination (MMSE) and Clock drawing test. Depression was examined with Montgomery and Aasberg Depression Rating Scale. Neurological examination, blood tests and structural brain imaging (CT) were performed. The diagnosis of dementia was set by the GP and discussed with at least one specialist in geriatric medicine according to the ICD-10 criteria as previously described^6^. Disagreement or uncertainty about the diagnostic subtypes regarding 12 patients was solved by consulting a third specialist in geriatric medicine. The controls were healthy age-matched individuals with normal cognitive function.

The **Oslo Parkinson’s disease Cohort Study** (OsloPD) recruited normal controls among spouses to participants diagnosed with PD, and from social clubs and among patients in general practice in the Oslo area. The normal controls were without any sign of neurological diseases, all were >40 years of age, had no known parkinsonism or other neurodegenerative disorder.

**Progression of Alzheimers Disease and Resource study** (PADR) recruited 222 patients at the outpatient clinic at the Department of Geriatric Medicine, St. Olav’s Hospital. These were home-dwelling patients assessed for cognitive impairment according to the ICD-10 research criteria^5^. Mainly patients diagnosed with Alzheimer’s disease (both mild cognitive impairment and dementia) were recruited. Recruitment for the PADR study was finished when this outpatient clinic joined the NorCog network and recuited patients to DemGene as part of NorCog. The PADR study focuses on different factors of importance for progression of Alzheimer’s disease with focus on depression, inflammation and cardiovascular diseases.

**TrønderBrain.** Patients were recruited through the University Hospital in Trondheim, the district hospital in Namsos, nursing homes, and local care authorities in central Norway. Patients or suitable proxies were asked about case history, including a family history of dementia. Neurological examination was completed by one single neurologist (SBS), and included the Mini Mental State Examination (MMSE) and the Clock Drawing Test. Blood screening was performed, and secondary causes of dementia were excluded. Additional tests including EEG, MRI and lumbar puncture were performed in subgroups. Further details are provided elsewhere^7^. Control subjects were caregivers not genetically related to the patients and other elderly volunteers recruited from societies for retired people in central Norway. All controls were without first-degree relatives with dementia, were healthy for their age and displayed no signs of a neurological disorder. They were age and sex-matched to the patient groups as closely as possible. For both patients and controls, only ethnic Norwegians were included.

**Iceland^8^**

Study Participants Icelandic Population Approval for these studies was obtained from the National Bioethics Committee and the Icelandic Data Protection Authority. Written informed consent was obtained from all participants or their guardians before blood samples were drawn, and all sample identifiers were encrypted in accordance with the regulations of the Icelandic Data Protection Authority. In 1062 patients, the diagnosis of Alzheimer’s disease was established according to the criteria for definite, probable, or possible Alzheimer’s disease of the National Institute of Neurological and Communicative Disorders and Stroke and the Alzheimer’s Disease and Related Disorders Association (NINCDS-ADRDA). In another 2697 patients, the diagnosis was established according to the criteria for code F00 of the International Classification of Diseases, 10th Revision (ICD-10). We assessed cognitive function using data from the Resident Assessment Instrument (RAI), with which assessment is performed on an individual basis and recorded in a Minimum Data Set (MDS 2.0) form. Data were primarily obtained through RAI 2.0 for Nursing Homes, which is a comprehensive and standardized instrument originally developed for residential facilities for the elderly, with additional information provided by the InterRAI Assessment for Home Care. We assessed cognitive function using the MDS Cognitive Performance Scale (CPS), which combines selected MDS 2.0 items expressing different measures of cognitive function on a seven-category scale, ranging from 0 (intact) to 6 (severe impairment). The CPS is hierarchical and based on an assessment of several measures of cognitive function; a 1-unit change is a reflection of distinct and measurable changes in at least one cognitive domain. A total of 1236 study participants with a score of 0 on the CPS scale were used as cognitively intact controls. We selected 110,050 population controls from among participants in various research projects at deCODE Genetics, excluding those in whom Alzheimer’s disease had been diagnosed. Association testing was carried out using information from 3,674 AD patients and 163,815 population controls.^9^

**References**

1. Auning E, Kjaervik VK, Selnes P, et al. White matter integrity and cognition in Parkinson's disease: a cross-sectional study. *BMJ Open*; **4**(1): e003976.

2. Selnes P, Aarsland D, Bjornerud A, et al. Diffusion tensor imaging surpasses cerebrospinal fluid as predictor of cognitive decline and medial temporal lobe atrophy in subjective cognitive impairment and mild cognitive impairment. *J Alzheimers Dis*; **33**(3): 723-36.

3. Aarsland D, Rongve A, Nore SP, et al. Frequency and case identification of dementia with Lewy bodies using the revised consensus criteria. *Dement Geriatr Cogn Disord* 2008; **26**(5): 445-52.

4. Bergh S HJ, Gabin J, Stordal E, Fikseaunet A, Selbæk G, Saltvedt I, Langballe EM, Tambs K. . Cohort profile: the Health and Memory Study (HMS): a dementia cohort linked to the HUNT study in Norway. *Int J Epidemiol* 2014; **43(6)**: 1759-68.

5. WHO. The ICD-10 classification of mental and behavioural disorders: clinical descriptions and diagnostic guidelines: World Health Organization; 1992.

6. Andersen F, Engstad TA, Straume B, et al. Recruitment methods in Alzheimer's disease research: general practice versus population based screening by mail. *BMC Med Res Methodol*; **10**: 35.

7. Sando SB, Melquist S, Cannon A, et al. APOE epsilon 4 lowers age at onset and is a high risk factor for Alzheimer's disease; a case control study from central Norway. *BMC Neurol* 2008; **8**: 9.

8. Jonsson T, Stefansson H, Steinberg S, et al. Variant of TREM2 Associated with the Risk of Alzheimer's Disease. *New England Journal of Medicine* 2013; **368**(2): 107-16.

9. Steinberg S, Stefansson H, Jonsson T, et al. Loss-of-function variants in ABCA7 confer risk of Alzheimer's disease. *Nat Genet* 2015; **47**(5): 445-7.

10. Fladby T, Pålhaugen L, Selnes P, et al. Detecting at-risk Alzheimer’s disease cases. *Journal of Alzheimer's Disease* 2017; **60**(1): 97-105.

11. Espinosa A, Alegret M, Valero S, et al. A longitudinal follow-up of 550 mild cognitive impairment patients: evidence for large conversion to dementia rates and detection of major risk factors involved. *Journal of Alzheimer's Disease* 2013; **34**(3): 769-80.

## Supplementary Table 1. Sample origins of DemGene

|  | **AD** | **MCI** | **Cases** | **#Women** | **Age** | **Controls** | **#Women** | **Age** |
| --- | --- | --- | --- | --- | --- | --- | --- | --- |
| **AHUS** | 125 | 113 | 238 | 121 | 69.03 ±9.04 | 77 | 40 | 65 ±9.49 |
| **COGNORM** |  |  |  |  |  |  |  |  |
| **DDI** | - | 89 | 89 | 41 | N/A | 93 | 60 | N/A |
| **DEMVEST** | 94 | 7 | 101 | 70 | 75.04 ±7.48 | 2 | 0 | N/A |
| **HUNT** | 530 | 44 | 574 | 400 | 75.01 ±8.40 | 128 | 74 | N/A |
| **NorCog** | 618 | 419 | 1037 | 552 | 69.79 ±10.21 | 90 | 50 | N/A |
| **NordNorge** | 148 | - | 148 | 89 | 80.53 ±6.91 | 193 | 79 | 72.65 ±5.40 |
| **OsloPD** | - | - | - | - | - | 443 | 193 |  |
| **PADR** | 124 | 31 | 155 | 98 | 76.59 ±6.52 | - | - | - |
| **TRONDER-BRAIN** | 496 | 55 | 501 | 366 | 74.93 ±10.11 | 634 | 373 | 73.24 ±9.13 |
| **Total Demgene** | 2,135 | 758 | 2,893 | 1737 (60.0%) | 73.19 ±9.91 | 1,660 | 869 (52.3%) | 72.15 ±8.90 |
| **Population controls** |  |  |  |  |  | 5,198 |  | 18-65 |
| **Total Demgene** | 2,135 | 758 | 2,893 | 1737 (60.0%) | 73.19 ±9.91 | 6,858 |  |  |

## Supplementary Table 2. Lead SNPs in the discovery sample

| GenomicLocus | uniqID | rsID | chr | pos | p | start | end | nSNPs | nGWASSNPs | nIndSigSNPs |
| --- | --- | --- | --- | --- | --- | --- | --- | --- | --- | --- |
| 1 | 1:207692049:A:G | rs6656401 | 1 | 207692049 | 3.83E-24 | 207302548 | 207900071 | 206 | 39 | 1 |
| 2 | 2:127892810:C:T | rs6733839 | 2 | 127892810 | 6.12E-47 | 127681156 | 127896213 | 331 | 99 | 4 |
| 3 | 2:234068476:C:T | rs35349669 | 2 | 234068476 | 8.70E-09 | 233966525 | 234116965 | 250 | 33 | 1 |
| **4** | **4:11718998:G:T** | **rs13133131** | **4** | **11718998** | **8.16E-09** | **11556893** | **11766157** | **401** | **203** | **1** |
| 5 | 6:47487762:A:G | rs10948363 | 6 | 47487762 | 1.06E-10 | 47312788 | 47708879 | 494 | 106 | 1 |
| 6 | 7:37883793:C:T | rs6966331 | 7 | 37883793 | 2.04E-08 | 37561227 | 37927550 | 438 | 108 | 1 |
| 7 | 7:99971834:A:G | rs1859788 | 7 | 99971834 | 2.31E-11 | 99207876 | 100240771 | 366 | 43 | 1 |
| 8 | 7:143109208:G:T | rs75045569 | 7 | 143109208 | 5.31E-15 | 143055029 | 143865789 | 201 | 51 | 3 |
| 9 | 8:27462481:A:G | rs7982 | 8 | 27462481 | 3.26E-26 | 26743231 | 27522576 | 461 | 116 | 4 |
| 10 | 10:11720308:A:G | rs7920721 | 10 | 11720308 | 4.84E-10 | 11492718 | 11728625 | 90 | 8 | 1 |
| 11 | 11:47695840:A:G | rs11039332 | 11 | 47695840 | 9.31E-10 | 46699124 | 48695777 | 907 | 222 | 1 |
| 12 | 11:59923508:A:G | rs983392 | 11 | 59923508 | 1.84E-17 | 59812483 | 60105199 | 521 | 245 | 1 |
| 13 | 11:85867875:A:G | rs10792832 | 11 | 85867875 | 3.60E-29 | 85541630 | 85900117 | 577 | 189 | 1 |
| 14 | 11:121435587:C:T | rs11218343 | 11 | 121435587 | 2.41E-16 | 121409219 | 121517613 | 39 | 27 | 1 |
| 15 | 14:53298277:A:G | rs12586707 | 14 | 53298277 | 2.82E-10 | 52785124 | 53795829 | 222 | 75 | 1 |
| 16 | 14:92931737:A:G | rs941648 | 14 | 92931737 | 1.25E-11 | 92840878 | 92969946 | 269 | 34 | 2 |
| **17** | **14:107164622:A:T** | **rs78631692** | **14** | **107164622** | **2.61E-08** | **106478028** | **107265510** | **715** | **16** | **1** |
| 18 | 17:56409089:C:G | rs2632516 | 17 | 56409089 | 3.58E-08 | 56328659 | 57262244 | 490 | 9 | 1 |
| 19 | 19:1063443:A:G | rs4147929 | 19 | 1063443 | 1.66E-14 | 1003070 | 1097240 | 223 | 40 | 3 |
| 20 | 20:55018260:C:T | rs7274581 | 20 | 55018260 | 1.65E-09 | 54979171 | 55117530 | 101 | 15 | 1 |

## Supplementary Table 3. Lead SNPs based on the combined sample

##

**Suggestive associations not discovered in IGAP**

|  |  |  |  |  |  | IGAP | Discovery | | Replication | |  | Combined | |
| --- | --- | --- | --- | --- | --- | --- | --- | --- | --- | --- | --- | --- | --- |
| CHR | BP | SNP | A1 | A2 | nearestGene | P | OR | P | OR | P | Dir. | OR | P |
| 1 | 193648828 | rs12138394 | T | G | ENSG00000226640 | 4.98E-07 | 1.09 | 9.41E-08 | 1.02 | 3.93E-01 | +++ | 1.07 | 4.21E-07 |
| 2 | 234003359 | rs7419666 | C | T | INPP5D | 3.14E-07 | 1.07 | 9.36E-08 | 1.04 | 9.36E-02 | +++ | 1.06 | 4.01E-08 |
| 4 | 11718998 | rs13133131 | T | G | ENSG00000249631 | 4.54E-07 | 1.07 | 6.88E-08 | 1.05 | 3.19E-02 | +++ | 1.07 | 8.16E-09 |
| 8 | 95965695 | rs13257021 | G | A | NDUFAF6 | 1.70E-07 | 1.06 | 5.56E-07 | 1.04 | 6.79E-02 | +-+ | 1.06 | 1.32E-07 |
| 10 | 11720308 | rs7920721 | G | A | ENSG00000271046 | 2.89E-07 | 1.07 | 1.82E-08 | 1.06 | 7.23E-03 | +++ | 1.07 | 4.84E-10 |
| 14 | 107164622 | rs78631692 | T | A | IGHV1-68 | 5.51E-07 | 0.89 | 2.61E-08 | 0.96 | 1.78E-01 | --- | 0.91 | 6.57E-08 |
| 15 | 59045774 | rs593742 | G | A | ENSG00000259173 | 1.41E-06 | 0.94 | 5.22E-07 | 0.95 | 4.07E-02 | --- | 0.94 | 7.58E-08 |
| 17 | 56409089 | rs2632516 | C | G | MIR142 | 8.79E-07 | 0.93 | 3.65E-09 | 0.99 | 5.11E-01 | --- | 0.94 | 3.58E-08 |
|  |  |  |  |  |  |  |  |  |  |  |  |  |  |

**Suggestive associations discovered in IGAP**

|  |  |  |  |  |  | IGAP | Discovery | | Replication | |  | Combined | |
| --- | --- | --- | --- | --- | --- | --- | --- | --- | --- | --- | --- | --- | --- |
| CHR | BP | SNP | A1 | A2 | nearestGene | P | OR | P | OR | P | Dir. | OR | P |
| 1 | 207692049 | rs6656401 | A | G | CR1 | 5.69E-24 | 1.17 | 3.83E-24 | 1.12 | 5.03E-05 | +++ | 1.16 | 2.17E-27 |
| 2 | 127892810 | rs6733839 | T | C | BIN1 | 6.94E-44 | 1.21 | 6.12E-47 | 1.15 | 4.09E-10 | +++ | 1.20 | 1.01E-54 |
| 6 | 47431284 | rs9473117 | C | A | ENSG00000266330 | 5.56E-11 | 1.09 | 1.17E-10 | 1.08 | 2.37E-03 | +++ | 1.09 | 1.09E-12 |
| 7 | 37841534 | rs2718058 | G | A | EPDR1 | 4.76E-09 | 0.93 | 2.25E-08 | 0.95 | 4.40E-02 | --- | 0.94 | 4.08E-09 |
| 7 | 99971834 | rs1859788 | A | G | PILRA | 6.91E-10 | 0.91 | 2.31E-11 | 0.96 | 9.65E-02 | --- | 0.92 | 2.67E-11 |
| 7 | 143110762 | rs11771145 | A | G | EPHA1-AS1 | 1.12E-13 | 0.91 | 2.01E-14 | 0.96 | 1.18E-01 | --- | 0.92 | 8.38E-14 |
| 8 | 27462481 | rs7982 | A | G | CLU | 4.08E-25 | 0.87 | 3.26E-26 | 0.95 | 2.29E-02 | --- | 0.89 | 3.73E-25 |
| 11 | 47380340 | rs3740688 | G | T | SPI1 | 1.42E-08 | 0.93 | 2.79E-08 | 0.93 | 1.18E-03 | --- | 0.93 | 1.28E-10 |
| 11 | 59923508 | rs983392 | G | A | ENSG00000254952 | 6.14E-16 | 0.90 | 1.84E-17 | 0.87 | 1.17E-09 | --- | 0.89 | 2.73E-25 |
| 11 | 85867875 | rs10792832 | A | G | RNU6-560P | 9.32E-26 | 0.87 | 3.60E-29 | 0.89 | 2.69E-07 | --- | 0.87 | 7.92E-35 |
| 11 | 121435587 | rs11218343 | C | T | SORL1 | 9.73E-15 | 0.77 | 2.41E-16 | 0.88 | 9.34E-02 | --- | 0.79 | 2.26E-16 |
| 14 | 53400629 | rs17125944 | C | T | FERMT2 | 7.95E-09 | 1.13 | 7.11E-09 | 1.12 | 1.50E-03 | +++ | 1.13 | 4.11E-11 |
| 14 | 92926952 | rs10498633 | T | G | SLC24A4 | 5.54E-09 | 0.91 | 4.44E-10 | 0.88 | 3.50E-06 | --- | 0.90 | 1.43E-14 |
| 19 | 1056492 | rs3752246 | G | C | ABCA7 | 1.39E-15 | 1.14 | 2.04E-14 | 1.02 | 4.02E-01 | +++ | 1.10 | 4.06E-12 |
| 20 | 55018260 | rs7274581 | C | T | CASS4 | 2.46E-08 | 0.87 | 1.65E-09 | 0.84 | 2.21E-05 | --- | 0.86 | 2.48E-13 |

## Supplementary Figure 1. Quantile-quantile plot of GWAS of Norwegian cohort and meta-analysis with IGAP.


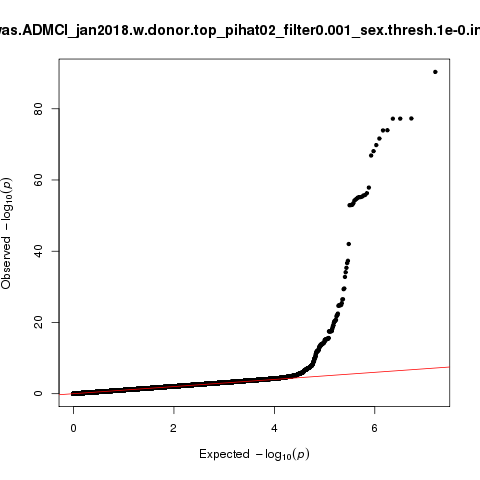


Quantile-quantile plot of the Norwegian cohort (inflation factor lambda = 1.022).


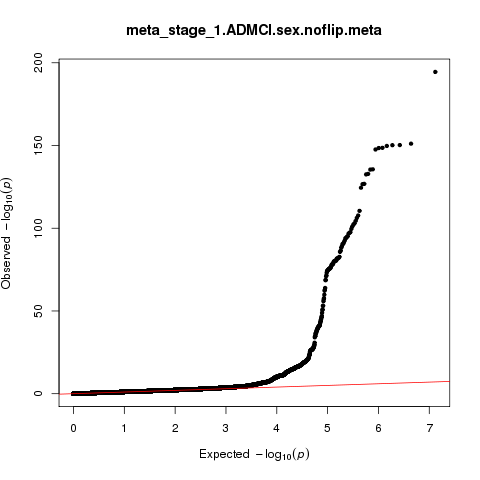


Quantile-quantile plot of the meta-analysis Norwegian cohort and IGAP (discovery sample).

## Supplementary Figure 2. Regional association plots on chromosome 10 and chromosome 17

**
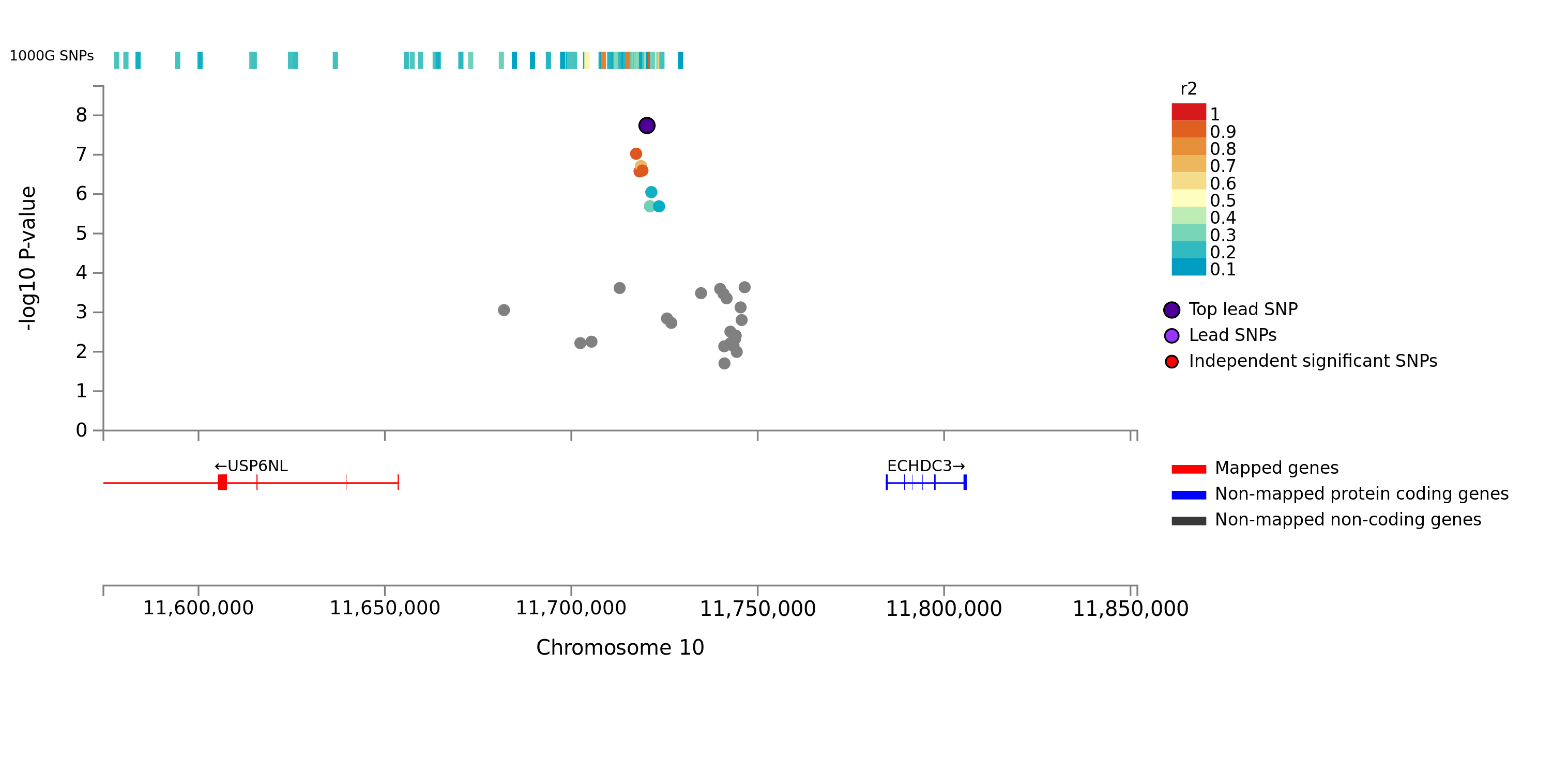
**

**
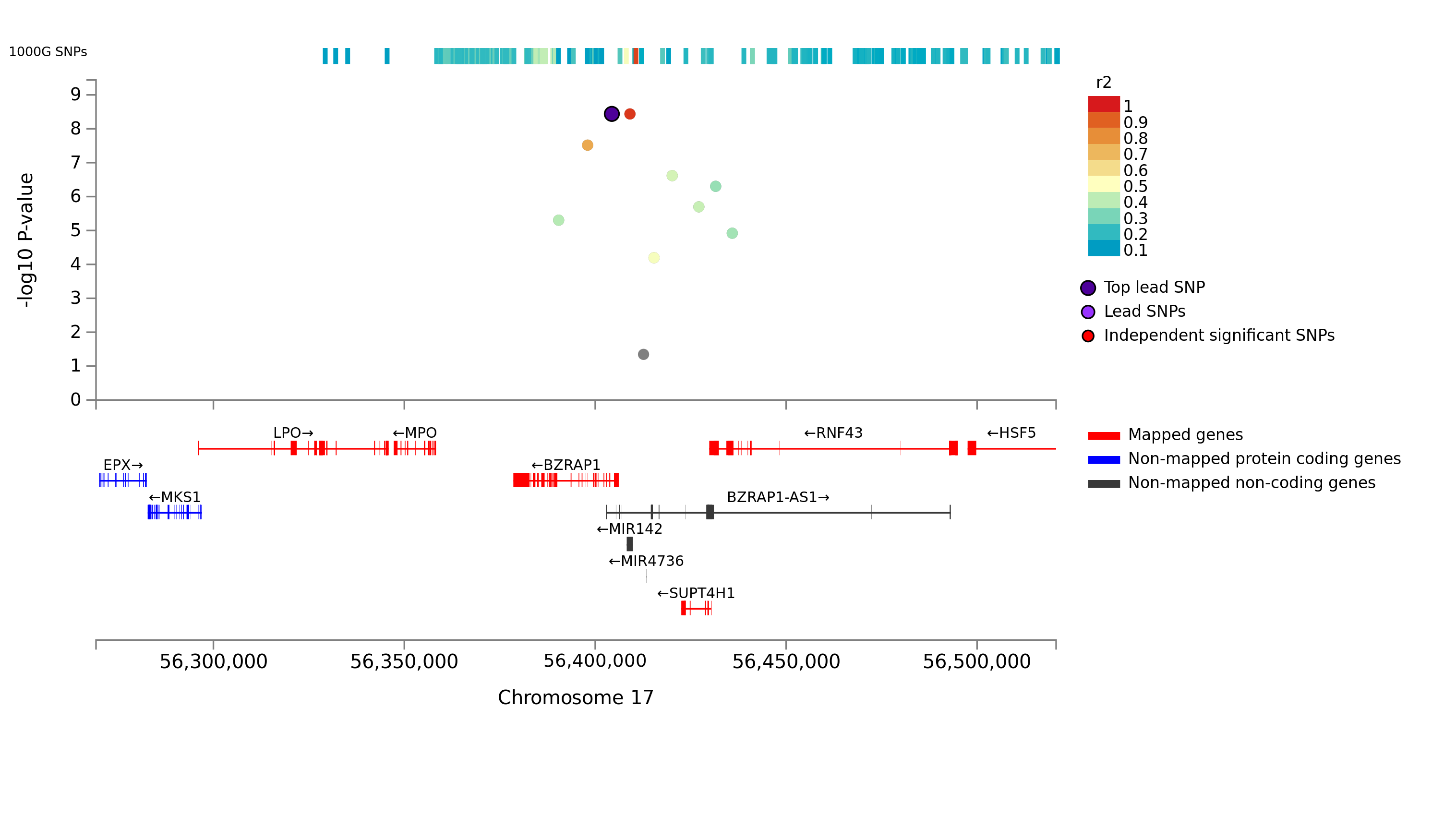
**

Regional association plots of top lead SNPs rs7920721 (chr 10) and rs2526378 (chr 17) in the discovery sample. The mapping of genes is based on position of the genomic risk loci.

## Supplementary Figure 3. FUMA summary of results


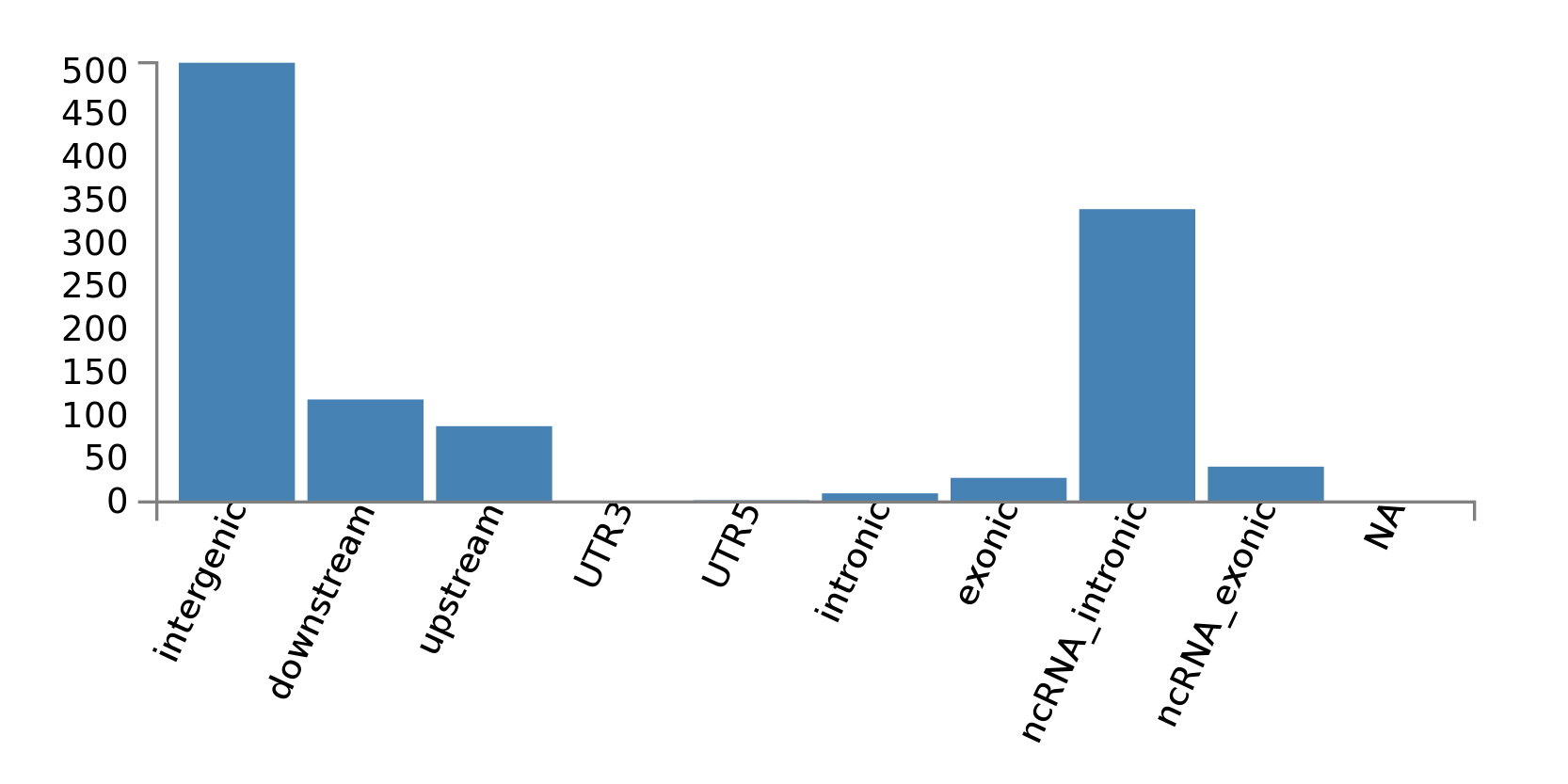


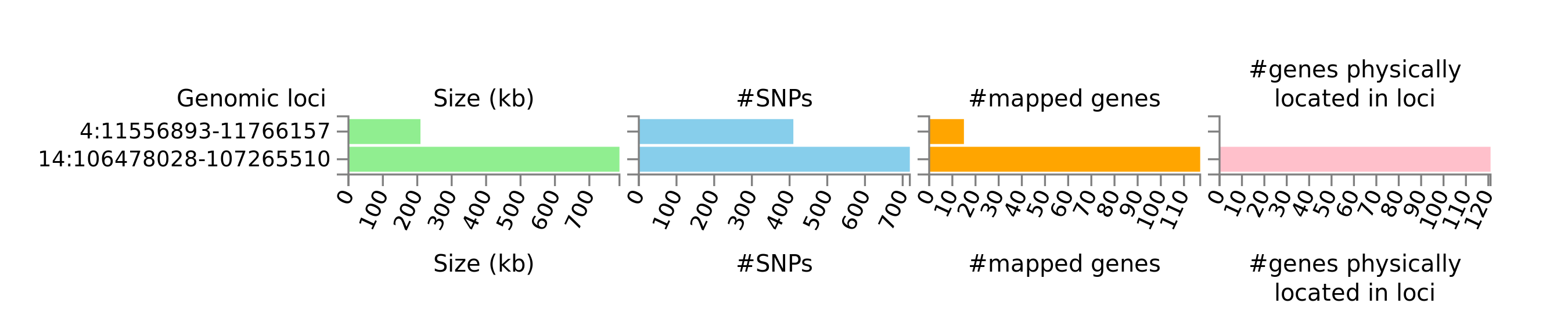


Top: Functional consequence of SNPs on genes. Out of 1129 SNPs in the loci at chromosome 4 and 14, 511 SNPs are intergenic and 340 SNPs are intronic non-coding RNA. Bottom: Summary per genomic risk locus, spanning 209kB in chromosome 4 and 787kB in chromosome 14. FUMA mapped 15 genes (8 protein-coding genes) to Chromosome 4 locus and 117 genes.

## Supplementary Figure 4. Chromatin interaction circos plot of chromosome 14 locus

**
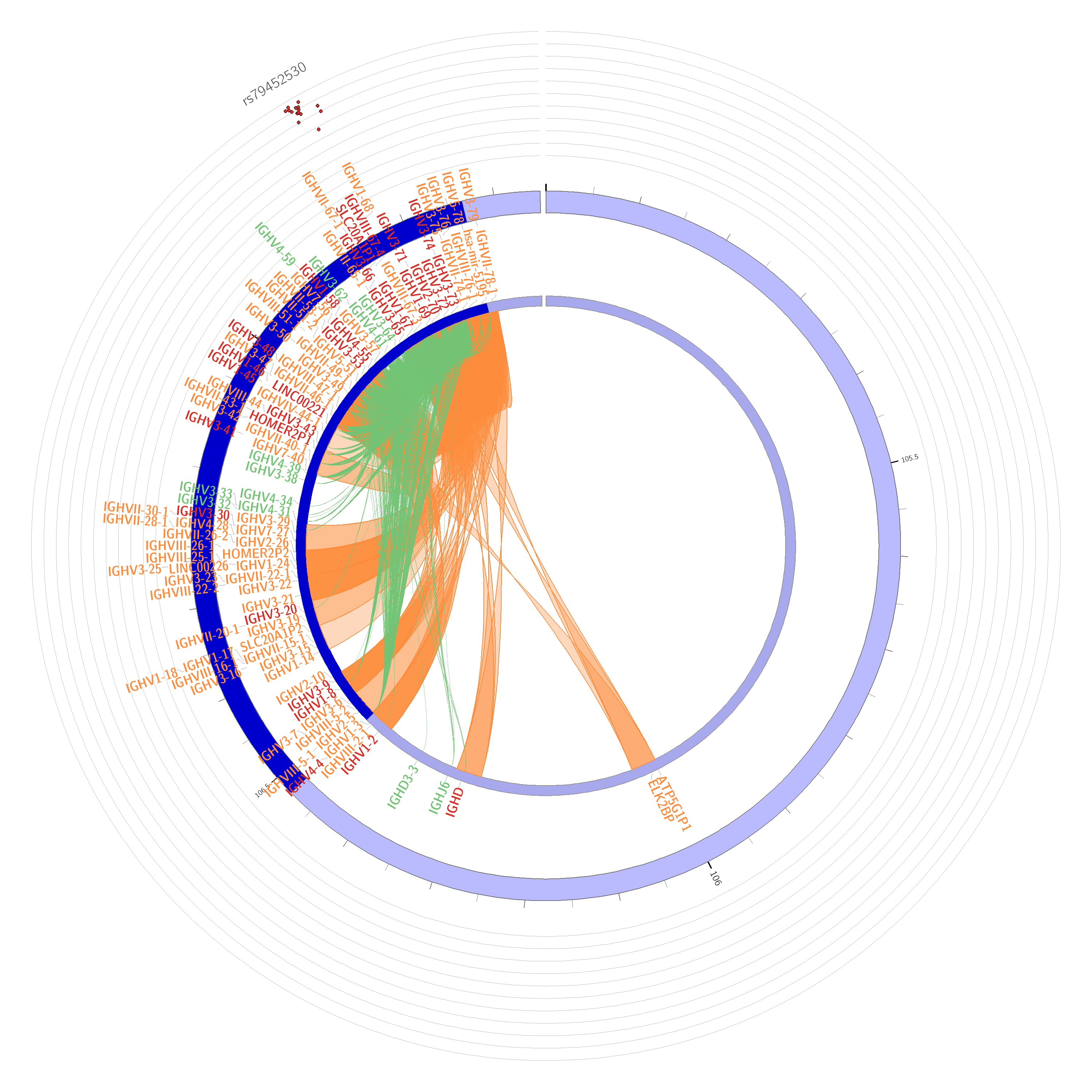
**

Circos plots of mapped gene on chromosome 14 locus. Genomic risk loci is highlighted in blue. Genes are mapped by 3-D chromatin interaction (orange) or eQTLs (green) or both (red).

## Supplementary Figure 5. Gene expression heatmap


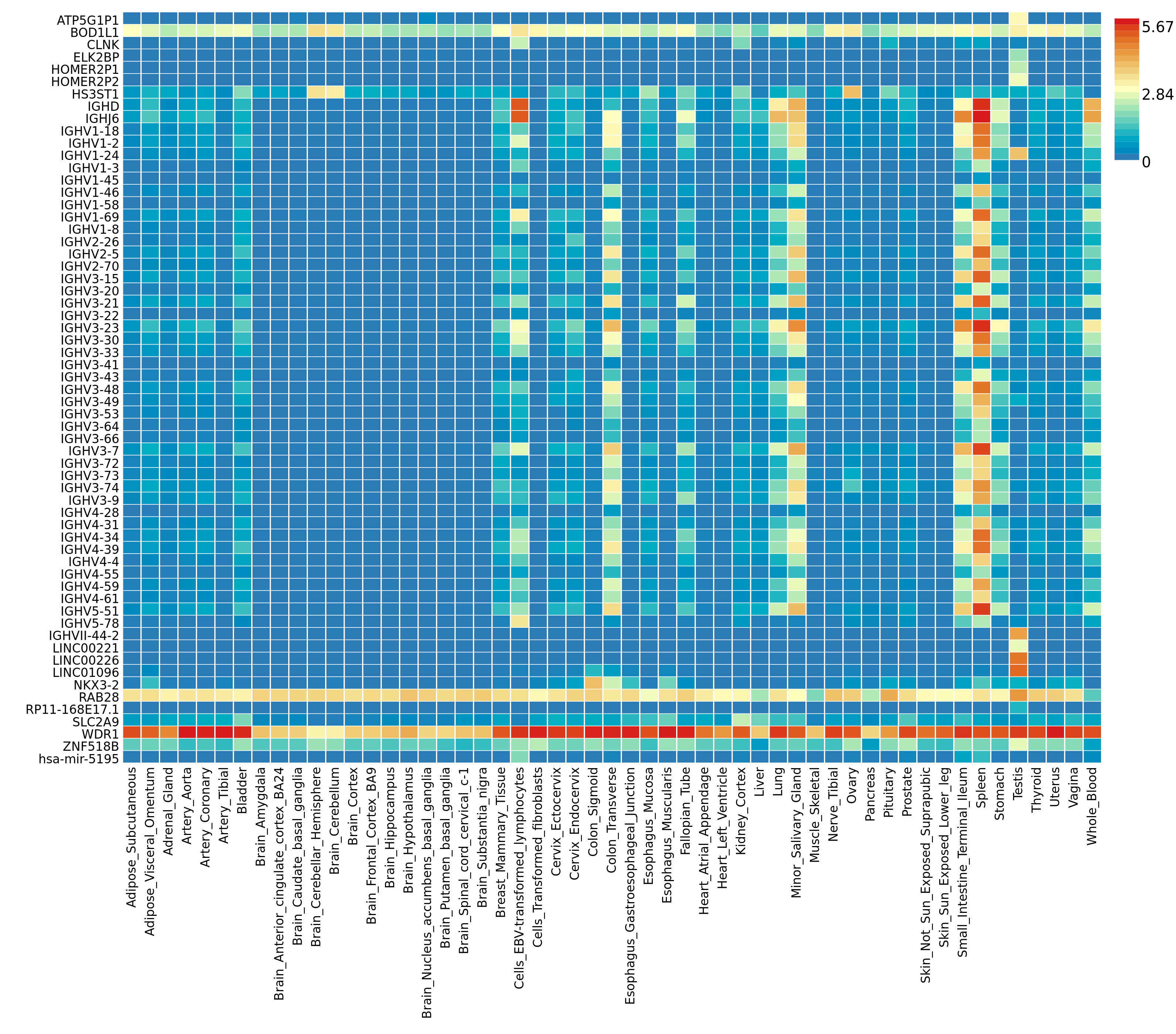
**Average expression per label**
This is an averaged expression value per label (e.g. tissue types or developmental stage) per gene following winsorization at 50 and log 2 transformation with pseudocount 1. The expression value depends on the data set, RPKM (Read Per Kilobase per Million) for GTEx v6 and BrainSpan, TPM (Transcripts Per Million) for GTEx v7. This allows for comparison across labels and genes. Hence, cells filled in red represent higher expression compared to cells filled in blue across genes and labels.


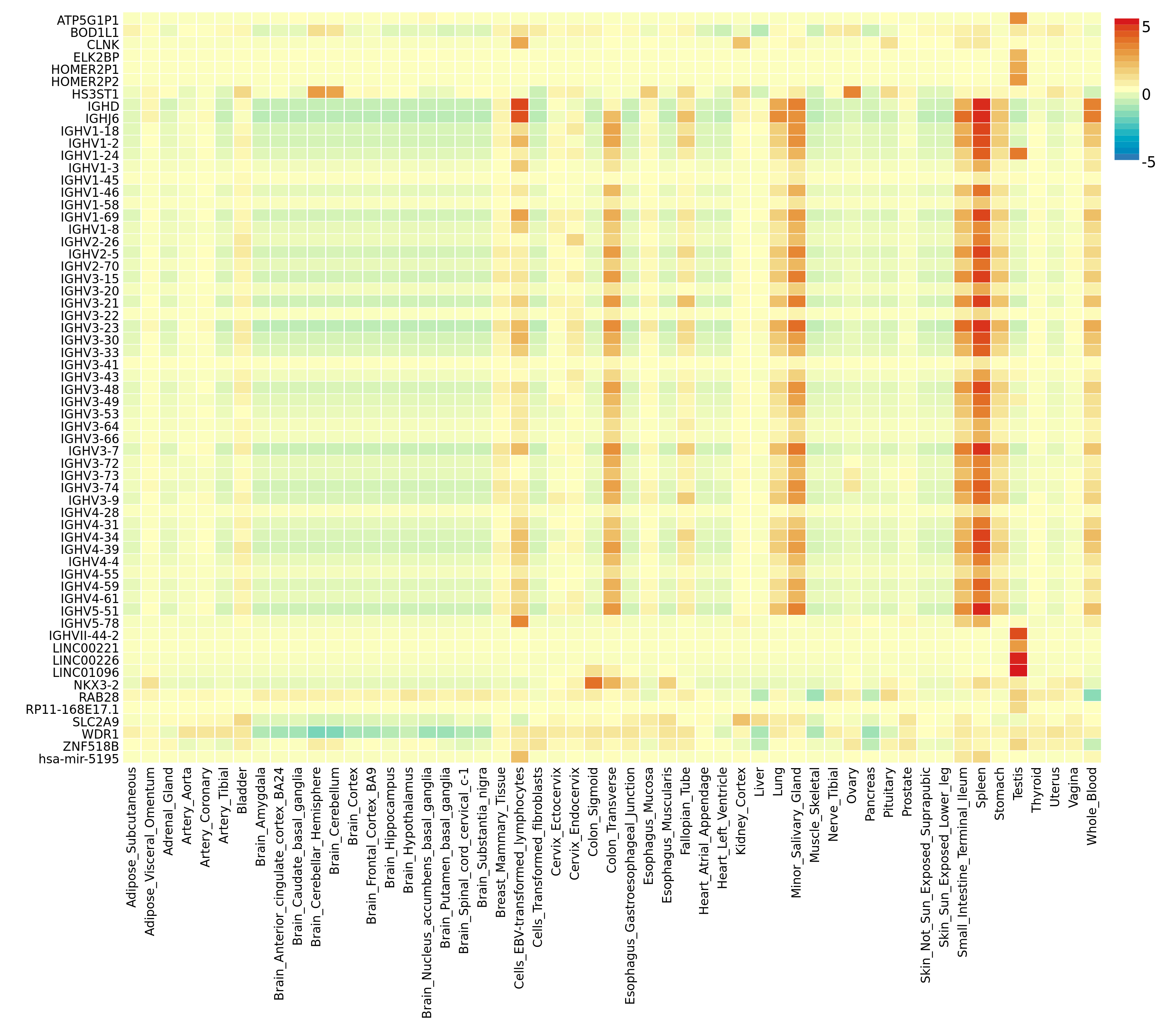


**Average of normalized expression per label**
This is the average of normalized expression (zero mean across samples) following winsorization at 50 and log 2 transformation of the expression value with pseudocount 1. This allows comparison of gene expression across labels (horizontal comparison) within a gene. Thus expression values of different genes within a label (vertical comparison) are not comparable. Hence, cells filled in red represents higher expression of the genes in a corresponding label compared to other labels, but it DOES NOT represent higher expression compared to other genes.
